# Potential biomarkers of aortic dissection based on expression network analysis

**DOI:** 10.1186/s12872-023-03173-3

**Published:** 2023-03-23

**Authors:** Junbo Feng, Yuntao Hu, Peng Peng, Juntao Li, Shenglin Ge

**Affiliations:** Department of Cardiovascular Surgery, The First Affiliated Hospital of Anhui Medical University, 218 Jixi Road, Hefei, Anhui 230000 People’s Republic of China

**Keywords:** Aortic dissection, miRNA, mRNA, GO analysis, PPI network

## Abstract

**Background:**

Aortic dissection (AD) is a rare disease with severe morbidity and high mortality. Presently, the pathogenesis of aortic dissection is still not completely clear, and studying its pathogenesis will have important clinical significance.

**Methods:**

We downloaded 28 samples from the Gene Expression Omnibus (GEO) database (Accession numbers: GSE147026 and GSE190635), including 14 aortic dissection samples and 14 healthy controls (HC) samples. The Limma package was used to screen differentially expressed genes. The StarBasev2.0 tool was used to predict the upstream molecular circRNA of the selected miRNAs, and Cytoscape software was used to process the obtained data. STRING database was used to analyze the interacting protein pairs of differentially expressed genes under medium filtration conditions. The R package "org.hs.eg.db" was used for functional enrichment analysis.

**Results:**

Two hundred genes associated with aortic dissection were screened. Functional enrichment analysis was performed based on these 200 genes. At the same time, 2720 paired miRNAs were predicted based on these 200 genes, among which *hsa-miR*-650, *hsa-miR*-625-5p, *hsa-miR*-491-5p and *hsa-miR*-760 paired mRNAs were the most. Based on these four miRNAs, 7106 pairs of circRNAs were predicted to be paired with them. The genes most related to these four miRNAs were screened from 200 differentially expressed genes (CDH2, AKT1, WNT5A, ADRB2, GNAI1, GNAI2, HGF, MCAM, DKK2, ISL1).

**Conclusions:**

The study demonstrates that miRNA-associated circRNA-mRNA networks are altered in AD, implying that miRNA may play a crucial role in regulating the onset and progression of AD. It may become a potential biomarker for the diagnosis and treatment of AD.

## Background

Aortic dissection (AD) is a cardiovascular disease with high mortality and risk; it is also one of the most challenging diseases in cardiovascular surgery [1]. The mortality rate increases by 1% to 2% per hour within 24 to 48 h and 75% within 2 weeks of onset if prompt untreated [2]. There are many influencing factors of AD, but hypertension is the leading cause of AD in China [3]. There are nearly 300 million hypertensive patients in China, and hypertension awareness and control rates are lower than those in developed countries [4]. Therefore, the potential incidence of AD in China is enormous. In recent years, the incidence of AD has been increasing yearly, and it is getting younger and younger [5]. With the continuous improvement of surgical diagnosis and treatment, surgical treatment is still one of the essential methods for the treatment of AD [6]. The surgical treatment methods include Sun's procedure, endovascular repair, hybrid surgery and covered stent intervention [7–9]. Although the postoperative survival rate of patients is greatly improved, surgical treatment is only a palliative treatment [7]. The pathological process of the aortic wall in AD patients will not be terminated by partial aortic resection, and the residual false lumen will face a lifelong risk of long-term neoplasia and rupture [10]. Studies have shown that the incidence of long-term postoperative complications in AD patients is still high, such as new hairpin layer [11], aneurysm formation or rupture [12], endleakage [13], stent displacement and stent rupture, which seriously affect the quality of life of patients. Therefore, long-term and even lifelong early prevention after surgery are significant.

Aortic dissection is rapid, ferocious and has a high mortality rate [14]. Early diagnosis of aortic dissection is crucial since most patients wait until the onset of the disease, when mortality is significantly increased and the disease is often misdiagnosed when it first occurs [15]. Most current studies focus on the treatment and pathogenesis of aortic dissection, but there are few studies on the markers of aortic dissection. In recent years, more and more studies have shown that miRNAs negatively regulate protein translation by binding to complementary mRNA sequences. Zhang et al. found that lncRNA-miRNA-mRNA ceRNA regulatory network plays a regulatory role in the occurrence and development of AD [16]. Liu and colleagues found that circRNA networks mediated by circRNAs may be novel biomarkers for aortic dissection [17].

In conclusion, lncRNA, miRNA, mRNA and ceRNA all impact the occurrence and development of aortic dissection, especially the network constructed by them is of great significance to aortic dissection. However, the effect of circRNA-miRNA-mRNA regulatory network on AD regulation is rarely reported. This study is a supplement to this finding, in order to explore the relationship between miRNA and AD.

## Methods

### Clinical samples of AD were retrieved from the GEO database

Use the GEO database screening of aortic dissection (AD) http://www.ncbi.nlm.nih.gov/GEO. The inclusion criteria were as follows: (1) the data set contained AD or healthy persons; (2) There are 6 or more specimens in the data set. Two eligible datasets were selected, including GSE147026 (expression profiling by high-throughput sequencing, GPL24676) and GSE190635 (expression profiling by array, GPL570). A total of 28 samples were collected from the data, including 14 AD and 14 healthy control (HC) samples. The RNA information of the selected samples was downloaded for further analysis. The sample information and data used in this part are downloaded from public databases and therefore do not require patient consent or ethics committee approval.

### The data processing

The original expression matrix is normalized. The Limma package was used to screen for differentially expressed genes. *P*-values for genes were calculated using the t-test, and adjusted *P*-values were calculated using the Benjamini and Hochberg methods. The following criteria selected differentially expressed genes: at least 1.0-fold change between healthy control and AD patient samples and adjusted *P*-value < 0.05.

### Analysis of enrichment

The gene names of differential genes (DEGs) are converted to gene ids by R package "org.hs.eg.db". Gene Ontology (GO) and Kyoto Encyclopedia of Genes and Genomes (KEGG) [18] analyses were implemented with the R "clustererProfiler" software package (version 3.14.3) to explore the possible functions of these DEGs further. Threshold *p* < 0.05 and Q-value & LT; 0.05 can screen out different GO terms and signal pathways. The results are visualized using R software packages RichhPlot and GGploT2.

### circRNA-miRNA-mRNA network

The TargetScan, DIANA-microT, miRDB, miRanda, Pita, MicroCosm, eimmo, PicTar databases were used to search for miRNAs that the mRNA might target. The predicted miRNAs that overlapped with at least three databases were selected as candidates. Then, the miRNA with the most binding difference mRNA was screened. On this basis, StarBase v2.0 tool is used to predict the upstream molecule circRNA of the selected miRNA, and Cytoscape software is used to process the obtained data to visualize the predicted results.

### Construction of protein–protein interaction network (PPI) and identification of Hub gene

STRING (https://STRING-db.org) was used to analyze the PPI network (score & GT; 0.4). Execute Perl to get the network files. The cellular Hubba plug-in of Cytoscape (V3.7.2) was used to score the top 10 algorithms for each node gene, namely maximal clique centrality (MCC), the density of maximum neighborhood component (DMNC), maximum neighborhood component (MNC). The five node genes with the highest score of the MCC algorithm were selected as screening genes for further analysis.

## Results

### Screening of differentially expressed genes

**Fig. 1:**
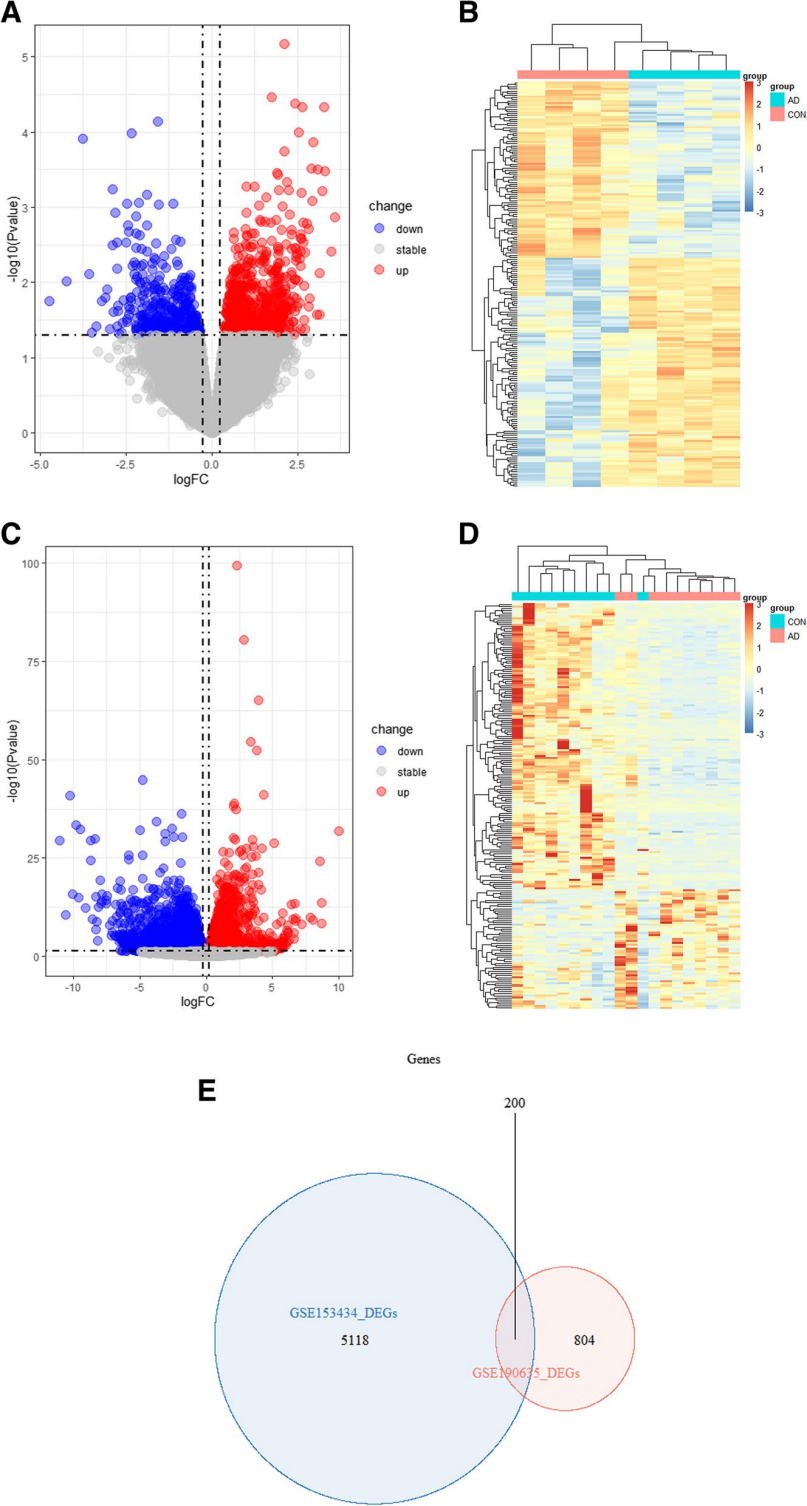
Differential gene analysis. **A**, **C** Volcano map showing significantly differentially expressed genes in AD and CON. Red dots represent raised genes, dot cut genes, said threshold for | log2FC |≥ 0.25 or higher, adjusted *p* < 0.05. Figure **A** is GSE147026, and Figure **C** is GSE190635 (**B**, **D**) heat map, showing the expressions of DEG in AD and CON. Darker red squares indicate higher DEG expression, while darker blue squares indicate lower DEG expression. Figure **B** is GSE147026, and figure **D** is GSE190635. **E** Venn diagram of overlapping DEGs from GSE147026 and GSE190635. A consensus of 200 overlapping mRNAs was identified

Two original datasets were studied, including 14 AD and 14 control groups (CON) selected for this study. Differential expression analysis of the data sets showed that compared with the control group, the DEG(GSE147026:total-2908/UP-1115/ Down-1793; GSE190635: total. 1004 / up. / 633 to the 371) significant differences of expression, the threshold for | log2FC |  ≥ 0.25 and *p* < 0.05. The expression of DEGs can be seen in the volcano map and heat map. Next, the expression of 200 genes between GSE147026 and GSE190635 was analyzed (Fig. 1).

### GO and KEGG enrichment analysis

**Fig. 2:**
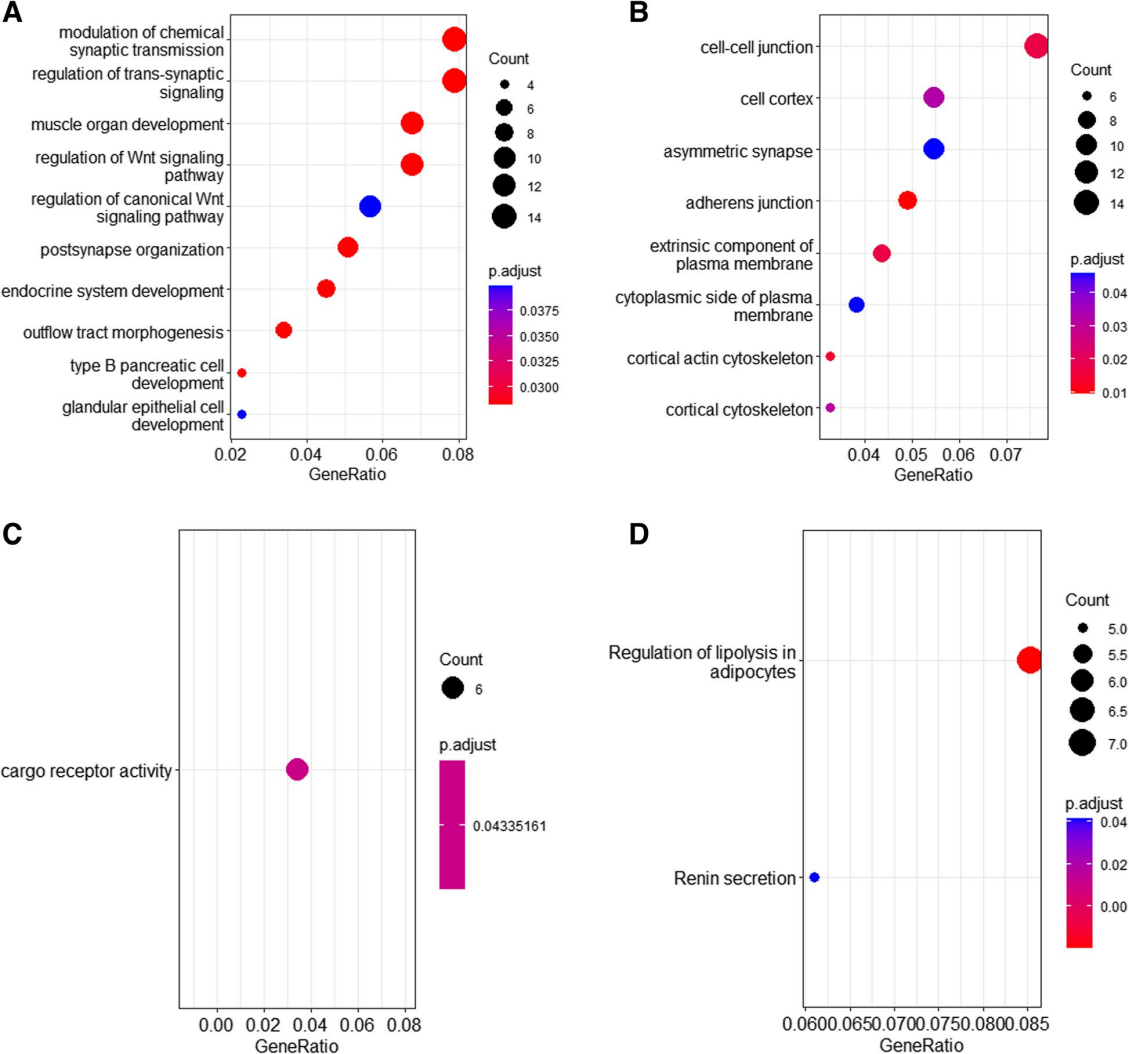
Results of GO and KEGG. Genomic (KEGG) pathway enrichment analysis of DEGs. **A ~ C** Top 5 genes in GO-BP, 5 genes in GO-CC and 1 gene in GO-MF analysis item in DEGs. **D** The first two most abundant KEGG pathways (including up-regulated and down-regulated pathways) of DEGs

The database was used for GO and KEGG enrichment analysis of 200 overlapping genes. For GO analysis, DEG was abundant in the "regulation of chemical synaptic transmission," "regulation of cross-synaptic signaling," and "muscle organ development" when Gene Ontology annotations of Biological Process (GO-BP) analysis was considered (Fig. 2A). The top three enrichment items for Gene Ontology annotations of Cellular Component (GO-CC) analysis were "cell–cell junctions," "cell cortices," and "asymmetric synapses" (Fig. 2B). For Ontology annotations of Molecular Function (GO-MF), the first enrichment item was "cargo receptor activity" (Fig. 2C). KEGG enrichment analysis showed that the two most critical pathways were "regulation of lipolysis in adipocytes" and "renin secretion" (Fig. 2D).

### Construction of miRNA-mRNA pairing, circRNA-miRNA pairing and circRNA-miRNA-mRNA network

**Fig. 3:**
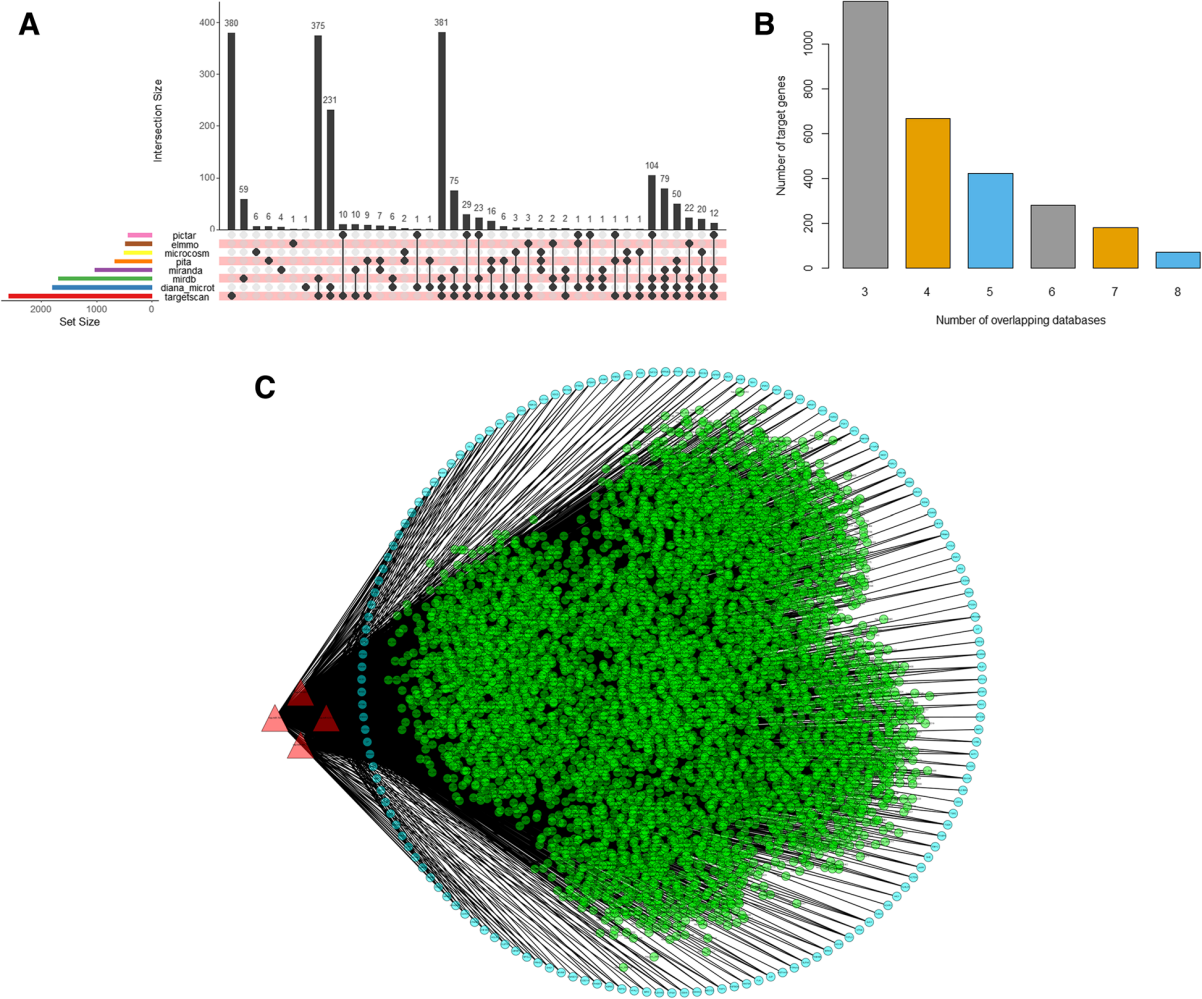
CircRNA-miRNA-mRNA Network construction (**A**) MultimIR tool with eight databases was used to clutter the predicted mRNAs of four miRNAs (miR-650, miR-625-5p, miR-491-5p and miR-760). **B** Show the number of overlapping genes from different databases. **C** CircRNA-miRNA-mRNA regulatory network, including 7106 circRNA nodes, 4 miRNA nodes, 155 mRNA nodes and 7483 disease edges. Red triangle: miRNA; Green node: circRNAs; Blue nodules: mRNA

Multimir predicted 2720 miRNAs across the eight databases using target gene overlap in at least three of the eight databases as the criterion (Fig. 3A, B). DEGs from GSE147026 and GSE190635 integrated with miRWalk target genes, and a total of 71,775 pairs of miRNA-mRNAs were identified. Among them, 97 mRNAs of *hsa-miR*-650, 95 mRNAs of *hsa-miR*-625-5p, 94 mRNAs of *hsa-miR*-491-5p and 91 mRNAs of *hsa-miR*-760 were obtained.

By applying the *starbase database* to identify the corresponding circRNA for each potential miRNA, 7106 circRNA-miRNA pairs were obtained. Specifically, 2120 circRNAs of *hsa-miR*-650, 1350 circRNAs of *hsa-miR*-625-5p, 1632 circRNAs of *hsa-miR*-491-5p and 2004 circRNAs of *hsa-miR*-760 have been identified. As shown in Fig. 3C, a circRNA-miRNA-mRNA network was preliminarily constructed based on miRNA-mRNA and miRNA-circRNA pairs consisting of 7106 circRNA nodes, 4 miRNA nodes, 155 mRNA nodes and 7483 edges (Fig. 3C).

### PPI Network analysis

A PPI network based on 200 overlapping DEGs associated with four miRNAs miR-650, miR-625-5p, miR-491-5p, and miR-760) was established using *Cytoscape* software(Version 3.7.2). The original network consists of 86 nodes and 94 edges. Using the *cytoHubba* plugin, 10 genes (CDH2, AKT1, WNT5A, ADRB2, GNAI1, GNAI2, HGF, MCAM, DKK2, ISL1) were identified in this cluster (Table 1).

**Table 1:** Hub gene list

**Rank**	**Name**	**Score**
1	CDH2	22
2	AKT1	21
3	WNT5A	13
4	ADRB2	8
5	GNAI1	6
5	GNAI2	6
5	HGF	6
8	MCAM	5
9	DKK2	4
9	ISL1	4

### Identify potential circRNA-miRNA-mRNA regulatory axes

**Fig. 4:**
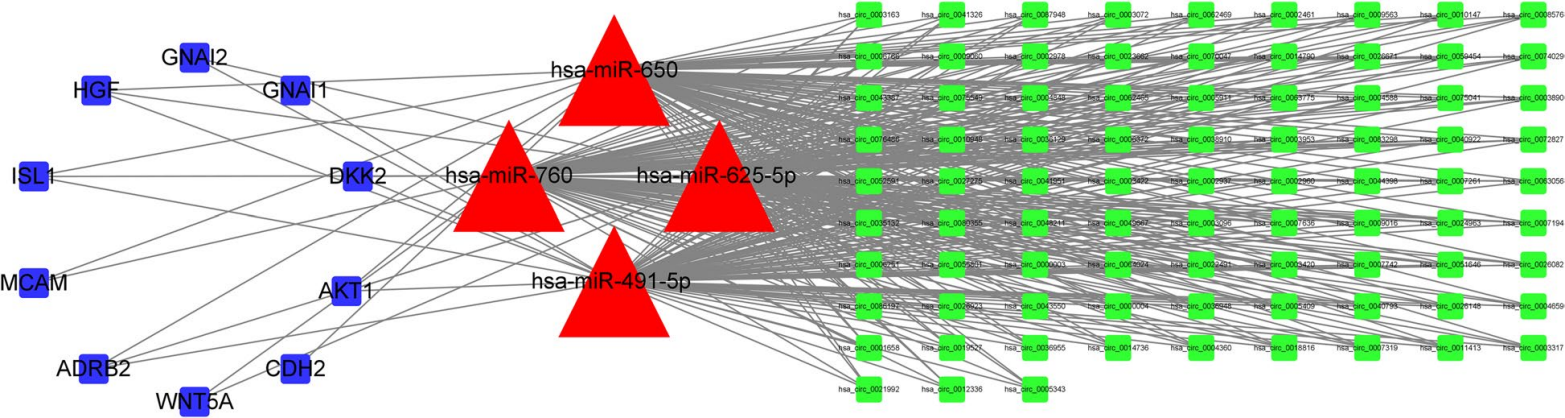
CircRNA-miRNA-mRNA regulatory network with 84 circRNA nodes, 4 miRNA nodes, 10 mRNA nodes and 358 disease edges. Red triangle: miRNA; Green node: circRNAs; Blue nodules: mRNA

After calculating the degree of circRNA in the preliminary circRNA-miRNA-mRNA network, circRNAs exhibited the highest degree (degree = 4). By extracting relevant miRNAs and mRNAs, a secondary net consisting of 98 nodes and 358 edges was constructed (Fig. 4).

## Discussion

Our study attempted to investigate the potential pathogenic mechanism of AD through a comprehensive analysis of two GEO datasets containing AD and healthy samples. We identified two modules with higher retention rates in the two datasets in the analysis results. In addition, the differential miRNAs in the two groups were identified, including *hsa-miR*-650,*hsa-miR*-625-5p, *hsa-miR*-491-5p and *hsa-miR*-760. We analyzed these four miRNAs to establish a PPI network and obtained 10 DEGs (CDH2, AKT1, WNT5A, ADRB2, GNAI1, GNAI2, HGF, MCAM, DKK2, ISL1) related to these four miRNAs.

CDH2 is overexpressed in cardiac, smooth muscle cells after myocardial infarction [19]. Transverse coarctation of the aorta occurs in hypertrophy and heart failure under pressure overload, and Akt1 hyperactivation occurs in left ventricular cardiomyocytes [20]. TAC enhanced the expression and secretion of WNT5A or WNT11 in cardiomyocytes (CM), cardiac fibroblasts (CF) and cardiac microvascular endothelial cells (CMEC) [21]. ADRB2 agonist promoted the activity of BDNF/TrkB and cAMP/PKA signaling pathways and alleviated HG-aggravated H/R injury in H9C2 cells.Caveolin-3 protects the diabetic heart from I/R injury through GnAI1/2, cAMP/PKA and BDNF/TrkB signaling pathways [22]. They are involved in regulating G protein-coupled receptor (GPCR) signaling. Previous studies have shown that the production of HGF in bone marrow stromal cells (BMSCs) leads to IL-11, IL-10, IL-6, IL-8, stromal cell-derived factor (SDF)-1α and vascular endothelial growth factor (VEGF) [23]. The expression of melanoma cell adhesion molecule (MCAM) is increased in abdominal aortic aneurysms [24]. METTL3 positively regulates PRI-MIR221/222 maturation in an M6A-dependent manner and subsequently promotes Ang-II-induced cardiac hypertrophy by inhibiting DKK2 activation of Wnt/β-catenin signaling [25].

Unlike miRNAs, circRNAs have high stability and tissue specificity, form a continuous covalent closed cycle, have no 5' or 3' polyadenylation tail, and are resistant to RNAser degradation or RNA exonuclide digestion. Recent evidence has linked circRNAs to a variety of human diseases, such as cancer [26], Alzheimer's disease [27], and cardiovascular disease [28]. However, no studies have shown that circRNA and the associated ceRNA network can be used as diagnostic or prognostic markers for AD.

More and more evidence has confirmed that circRNAs can act as "miRNA sponges" to inhibit miRNA inhibition of their target genes [29]. Of note, the mechanism of the circRNA effect on AD has not been investigated. However, previous studies have found differential circRNA expression prevalent between human AD tissues and normal control tissues. Therefore, we hypothesized that the dysregulation of ceRNA expression would affect the pathogenicity and progression of AD and chose circRNA as the entry point to study the underlying mechanism of miRNA.

Our study has some limitations that need to be addressed. First, all microarray datasets are obtained from purely public data, and there are some unavoidable biases, such as gender and age differences. In addition, only a small number of data sets were analyzed in this study. In addition, further examination of genes altered with AD is needed to determine whether overexpression/knockdown of these genes plays a vital role in AAD in vivo, which may also motivate novel therapeutic approaches.

## Conclusions

Our study demonstrates that circRNA-associated miRNA-mRNA networks are altered in AD, implying that circRNA may play a crucial role in regulating the onset and progression of AD. It may become a potential biomarker for the diagnosis and treatment of AD.

## Data Availability

The datasets generated and analyzed during the current study are available in the GEO database (accession numbers: GSE147026 and GSE190635) repository.
